# Well-Being and Throwing Speed of Women Handball Players Affected by Feedback

**DOI:** 10.3390/ijerph17176064

**Published:** 2020-08-20

**Authors:** Diego Soto, Juan Antonio García-Herrero, Rodrigo J. Carcedo

**Affiliations:** 1 Department Physical and Sport Education, University of León, 24007 León, Spain; dsotg@unileon.es; 2 Department of Didactics of Musical, Plastic and Corporal Expression, University of Salamanca, 37008 Salamanca, Spain; gherrero@usal.es; 3 Department of Developmental and Educational Psychology, University of Salamanca, 37005 Salamanca, Spain

**Keywords:** well-being, throwing speed, throwing accuracy, competence, motivation, feedback

## Abstract

This research aims at studying the effect of feedback on well-being (vitality, and positive and negative affect), competence valuation, perceived competence, motivation, and performance (throwing speed and accuracy) in a throwing task. Thirty nine expert women handball players, with experience in international handball competitions, participated in this study. They were indiscriminately ascribed to one of three different experimental conditions measuring feedback: (positive, negative, and none). Significant differences in well-being (positive affect) and throwing speed were found among the three feedback groups. More concretely, higher levels of positive affect and throwing speed were found in the negative feedback group in comparison with the other two groups (positive and no-feedback). These results have important implications for athletes’ well-being and performance, and for coaches’ training programs.

## 1. Introduction

Among the varied responsibilities that a coach takes on is the providing of information so that his or her athletes can progress in the execution of tasks. The effect of feedback, on both performance and motor learning, is no longer looked at solely as a means to improve performance. Rather, evidence is emerging suggesting that feedback provided to athletes is useful for learning motor skills [1] as well as having an effect on other variables that determine performance [2]. In this paper we present the results of a study regarding the impact that feedback has on different behavioral aspects in expert women players, such as motivation [3,4,5,6], motor learning [7,8], perceived competence [9,10,11]), self-efficacy and performance relationship [12], and well-being [13].

Studies related to a coach’s feedback and behavior have shown that training conditions which induce positive feelings related to participants’ results can increase perceived competence and self-efficacy as well as performance and motor learning [4,9,13,14]. In a study by Schunk and Cox [15], participants’ actual performance was irrelevant to the feedback that they received. The results revealed that feedback has a strong impact on self-efficacy. The study by Badami, VaezMousadi, Wulf and Namazizadeh [16], which provides feedback on accuracy of golf putting trials to a group of university students, indicated that feedback regarding the accuracy of their trials resulted in higher levels of self-confidence and effective learning. However, it is important to mention that all participants in this study presented higher levels of experience.

The coach’s influence is related to communication skills and type of feedback used [17]. Through their behaviors, they can have profound cognitive, behavioral, and emotional impact on their athletes too [18]. In this sense, the supportive behavior of a coach seems to act as an essential factor to achieve greater efficacy in expert women handball players [19]. The results of this study indicated that higher levels of perceived training and instructional behavior, positive feedback, along with social and instrumental support, predicted greater collective efficacy. In contrast, a higher perception of negative activation predicted lower levels of collective efficacy. In sum, these results highlight the relationship between coach behavior during training and competition, and their combined effect on collective effectiveness in expert women’s handball teams.

Within the eudaemonic perspective of well-being, vitality has been conceptualized as a psychological experience consisting of possessing enthusiasm and encouragement, which leads a person to a positive feeling of aliveness and energy [20]. While this aspect has been positively associated with the high perception of a coach’s positive feedback, no differences were found between mild and strong positive feedback [13]. At last, vitality has also been found to be indirectly associated with the type of feedback received, the perception of the coach’s feedback [8], and the coach’s autonomy support behaviors (a type of positive feedback) [21,22,23]. Individual’s perceived competence and autonomous motivation acted as mediators in the relationship between these three variables and vitality.

Positive feedback is identified by coach expressions emphasizing what has been done well throughout the task, praise, or athlete encouragement. Several studies including non-expert participants have explored the role of positive and negative feedback in sports tasks [8,24]. Positive feedback seems to be related to higher levels of intrinsic motivation and perceived competence [2,11,25]. In addition, several theories of motivation suggest that positive feedback is more effective in motivating oneself to pursue goals than negative feedback is because it increases both the expectation of reaching the goal, and an athlete’s self-efficacy [2,26,27]. In this sense, those participants who received positive feedback set higher goals than those who received negative feedback, whereas no differences in performance were observed [28]. In the same vein, different studies have confirmed that positive feedback is a key factor in improving self-efficacy [29,30,31].

Several studies suggest that positive feedback has an influential role in the perception of competence [9,11]. In a research study into young athletes of different sports, the use of positive feedback during training sessions was associated with a higher self-perception of competence, interest, and enjoyment [9]. In another study with female hockey players, positive and encouraging feedback after an error was shown to have a strong relationship with a high perception of competence and satisfaction in sport [14]. Furthermore, a study of inexperienced participants has provided evidence that positive feedback produces higher levels of perceived competence, autonomous motivation, and subjective vitality [32].

It seems that negative or change-oriented feedback [33] does not have a negative effect on athletes in all cases, considering variables such as a participants’ age or their level of experience and knowledge of the task [10,34,35,36]. For Weinberg and Gould [37], this feedback fulfills two important functions: firstly, offering information about the discrepancy between actual and desired performances; secondly, this feedback guides athletes to the specific changes they need to make if they want to improve upon future performance. Carpentier and Mageau [33] determined that change-oriented feedback can have positive results when administered properly, however, it is the way in which a coach administers the information that can determine its effect. Be that as it may, various studies agree that negative feedback can also have negative consequences on motivation, self-esteem, perceived self-efficacy, or on the coach–athlete relationship [30,38,39,40], and produce lower levels of collective efficacy [19].

The results concerning the effect of feedback on performance are less consistent. Differences in performance have not been found due to the administration of different types of feedback in some studies [13,28,41,42]. In this sense, it is possible that the type of feedback may have an effect on psychological variables (e.g., perceived competence and autonomous motivation), but not in sports performance. Some authors argue that motivation may have a positive effect on performance in the long run whereas the experimental tasks evaluate the immediate or short-term performance [13].

In this research, the main goal is to study the direct relationship between three types of feedback (positive, negative, and without) and its effects on competence valuation, perceived competence, autonomous motivation, and performance (throwing speed and accuracy) in a handball shooting task.

## 2. Materials and Methods

### 2.1. Participants

Thirty-nine expert female handball players from Chile’s national teams in the senior, junior, and youth categories participated in this study (age: Mean = 19.97 years, Standard Deviation (SD) = 3.60). All players had taken part in international matches and had more than eight years of experience. Each participant had experience in the concrete prescribed task. The participants were only informed that they were going to take part in a performance task and were randomly assigned to a feedback group: positive (*n* = 13), negative (*n* = 13), or lack of feedback (*n* = 13). All the norms of the Declaration of Helsinki were respected in this research. Additionally, all participants signed a consent form.

### 2.2. Apparatus and Task

**Figure 1:**
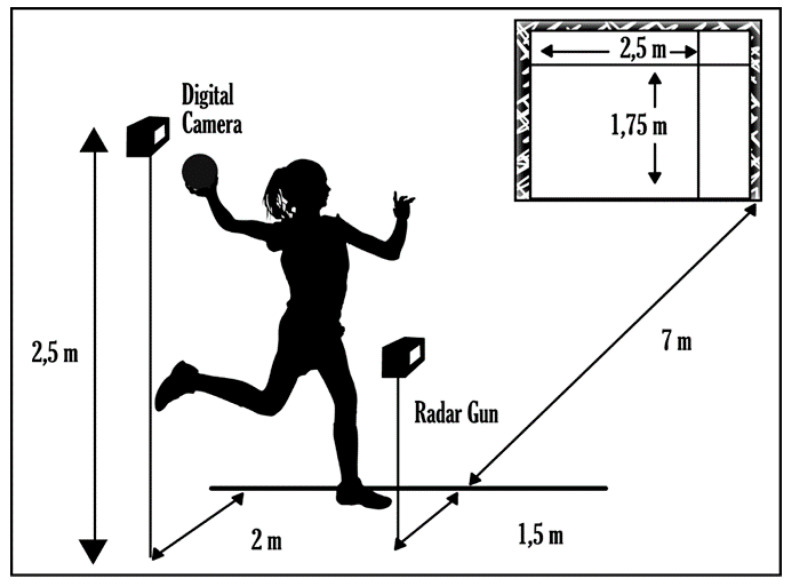
Graphical representation of the ball throwing task.

The selected task was very similar to that used in previous studies [43,44]. The participants had to throw a ball as forcefully and as accurately as possible. The goal was to hit a cross, seven meters from the launch area, which was created by stringing two rubber bands across a handball goal (3 m × 2 m) (Figure 1). An official ball from the International Handball Federation was used for this study (number 2, 56 cm in circumference, and mass 375 g).

### 2.3. Procedure

After warming up, each participant performed five standing maximal throws toward the goal with the only instruction of throwing as fast as possible in order to measure the maximum throwing speed. One minute of rest was given between each throw. The highest speed found within the five attempts was taken as the maximum throwing speed.

Participants were randomly assigned to three groups: positive feedback, negative feedback, or no-feedback, maintaining the same proportion of men and women in each group. Participants performed six sets of 5 pitches, resting 5 s between each throw, and 60 s per 10 pitches. In order to determine the rest time between each throw and series, the guidelines that Tripp, Boswell, Gansneder, and Shultz [45] had proposed in their work were adhered to. The instruction given to the participants was to throw as forcefully and as accurately as possible. The non-feedback group performed 30 pitches, receiving no information throughout the task. Positive and negative feedback groups received the first feedback after the 10th trial. From this point onwards, both groups received feedback every five pitches, regardless of their actual performance. Social-comparative and evaluative feedback was given: “With those pitches you are going to be among the best/worst” or “you are deviating very little/a lot, you are doing very well/pretty poorly” (positive/negative feedback groups). Feedback to all participants was provided by the same researcher (male). Both tone of voice and facial characteristics in giving feedback were consistent with the type of instruction given. Participants were utterly alone when performing the task. Participants were debriefed once the study had concluded, and were informed about the pre-set nature of the feedback received, which was not necessarily related to their actual performance.

A questionnaire assessing the psychological variables was completed by the participants before and after performing the ball throwing task.

### 2.4. Measures

#### 2.4.1. Independent Variable

Feedback. Three types of feedback were included in this study: positive, negative, and no-feedback were coded as follows: 0 for no-feedback, 1 for negative feedback, and 2 for positive feedback.

#### 2.4.2. Outcomes

##### Psychological Variables

(1) Competence Valuation

Competence valuation assesses the extent to which individuals value doing well on an upcoming task [46]. It was measured with a three-item scale: (Cronbach’s α = 0.91 before the feedback and Cronbach’s α = 0.83 after the feedback) and included the two items used by Elliot [47], “It is important to me to do well in this task” and “I care very much how well I do in this class”) as well as another item added by our research team (e.g., “It is very valuable for me to do this task well”). Participants needed to report their level of agreement with each of the three items on a 7-point Likert scale ranging from 1 (strongly disagree) to 7 (strongly agree).

(2) Perceived Competence

An adaptation of the five-items from the corresponding subscale of the Intrinsic Motivation Inventory [48] was utilized to evaluate participants’ competence perceptions toward the task (e.g., “I think I am pretty good at throwing”; Cronbach’s α = 0.80 before the feedback and Cronbach’s α = 0.85 after the feedback). Responses were given on a 7-point Likert scale that ranged from 1 (strongly disagree) to 7 (strongly agree).

(3) Autonomous Motivation

To measure this construct, an adaptation of the Spanish version of the Echelle de Motivation dans les Sports (EMS, Spanish version) [49], (original version) [50], was utilized [13]. The autonomous motivation subscale (Cronbach’s α = 0.92 before the feedback and Cronbach’s α = 0.95 after the feedback) was utilized by averaging the intrinsic (e.g., “Because I enjoyed it”) and identified motivation scores (e.g., “Because it is important to me”). Items were answered on a 7-point Likert-type scale, ranging from 1 (does not correspond) to 7 (corresponds exactly).

(4) Subjective Vitality

This variable was used as a measure of an individual’s well-being. An adaptation of the six-item version of the original Ryan and Frederick’s [20] Subjective Vitality Scale [51] was used (e.g., “I feel energized”; Cronbach’s α = 0.91 before the feedback and α = 0.94 after the feedback). Vitality assessed the extent to which participants felt energetic and active before and after the task of throwing. Answers were given on a 7-point Likert scale that ranged from 1 (strongly disagree) to 7 (strongly agree).

(5) Subjective Well-Being

This variable was measured across two dimensions: positive and negative affect. Both dimensions were assessed using the Spanish version of the Positive and Negative Affect Scale (PANAS) [52], original version [53]. A total of 20 items describing feelings and emotions, of which 10 describe a positive affect (e.g., enthusiasm) and another 10 items measure a negative affect (e.g., irritable). Good levels of reliability were obtained both in positive affect before (α = 0.83) and after the intervention (α = 0.90) and in the negative affect on both occasions (before: α = 0.87; after: α = 0.92).

For all scales, a total score was calculated adding the individual score of each item and dividing them by the number of items answered. Higher scores represented higher levels in a variable.

##### Performance Variables

(1) Throw Speed

To measure throw speed, the average maximum speed percentage was calculated by averaging the value of ten pitches. To calculate this percentage, the absolute value of the velocity t in km/h of each participant’s throw was divided by their maximum throwing speed, and then by multiplying the result by 100.

To measure an individuals’ maximum throwing speed, each participant performed five standing maximal throws toward the goal without any feedback regarding accuracy and with one minute of rest between each throw. The throw with the highest speed was chosen out of the five attempts.

Finally, the percentage of performance in relation to the maximum throwing speed was utilized to measure this variable. A radar gun was obtained from Sports Radar Ltd. (model SR 3600) to measure throwing speed.

(2) Throw Accuracy

A digital Panasonic SDR-H80 (Panasonic Corp., Osaka, Japan) camera was placed opposite the goal at a distance of 9 m from the goal line and at a height of 2.5 m. The center of the ball as it entered the goal was digitalized by the computer software “Kinovea©,” which identified the deviation of the throws with respect to the goal. The point at which the ball entered the goal was indicated digitally, and the coordinates of the real position were calculated (for deviation in both the X and Y axes) by using the dimensions of the goal as a reference. As described by Hancock, Butler, and Fischman [54], the mean radial error (MRE) was used to measure throwing accuracy. The MRE was measured as the average of the absolute distance to the center of the target of the ten pitches in sets 1, 2, and 3.

### 2.5. Statistical Analyses

Violations of normality and variance homogeneity in all repeated measures ANOVA models, the small sample size, and the use of ordinal Likert-type scales data, required a non-parametric approach [55,56,57]. Currently, there is a lack of adequate alternatives to parametric tests in the context of factorial designs when multivariate normality or equal covariance matrices across groups may not be assumed, or these tests do not allow for analysis of interaction effects across within-subject and between-subject variables. The parametric tests are unreliable or even false if these assumptions are not met or impossible to verify (Noguchi et al., 2012). However, in the context of repeated measures analyses, a non-parametric alternative for ANOVA analyses has been developed within the software package “nparLD” [58] included in “R 4.0.1”. For this study, the f1-ld-f1 function was used.

Post-hoc tests were run to interpret significant feedback*time of measure interactions. In this sense, between-group differences among feedback groups were analyzed with a post-hoc Tukey test using the function nparcomp. Post-hoc pairwise for within comparisons across the different times of measure were tested using a nonparametric studentized permutation analysis with 10,000 repetitions (function npar.t.test.paired) and a Bonferroni correction for multiple comparisons (the observed *p*-values for each comparison were multiplied by the number of comparisons). Both type of analyses were performed using the Rpackage ‘nparcomp’ [59]. At last, Cliff’s Delta was used to measure the nonparametric effect size of pairwise comparisons using the R package “effsize” [60].

All the analyses assumed an α-level of 0.05.

## 3. Results

A series of nonparametric ANOVA with one sub-plot factor (set of pitches or before–after the throwing task) and one whole-plot factor (feedback) were run in order to test the impact of these factors on psychological and performance variables. Descriptive data can be seen in Table 1.

In nonparametric repeated measures ANOVA, sub-plot and whole-plot factors refers to within-participant and between-participant factors respectively in parametric repeated measures parametric ANOVA.

With respect to performance variables, the interaction feedback*time of measure (1–10 pitches, 11–20 pitches, and 21–30 pitches) was found significant for throwing speed. Participants who received negative feedback showed higher levels of throwing speed at 11–20 and 21–30 pitches than at 1–10 pitches, meanwhile no differences were found in the other feedback groups (see Table 2).

Likewise, time main effects were determined for throwing speed and feedback main effects for throwing accuracy. On the one hand, independent of the type of feedback received, throwing speed was higher at 11–20 pitches (*p* < 0.01) and 21–30 pitches *(p* < 0.001) than 1–10 pitches. On the other hand, independent of the set of pitches, the positive feedback group showed a higher accuracy than the negative (*p* < 0.01) and the no-feedback (*p* < 0.01) groups.

Considering psychological variables, the interaction feedback*time of measurements (before and after receiving feedback) only resulted in being statistically significant for a positive affect. Post-hoc tests revealed that those who received negative feedback decreased their levels of positive affect, whereas those who received positive feedback or none, experienced no change in their levels of positive affect (see Table 3).

In addition, time effects were determined for competence valuation. Independent of the type of feedback received, higher levels of competence valuation were found before the task than after (*p* < 0.001).

## 4. Discussion

The purpose of this study was to investigate whether different types of feedback modified competence valuation, perceived competence, autonomous motivation, subjective vitality (as an indicator of well-being) and performance in expert women handball players. Based on previous studies [10,32], positive feedback could increase athletes’ perceived competence, although many of these studies have been developed with inexperienced participants. The results found in our work are not consistent with these previous findings given that there were no changes in perceived competence due to the type of feedback received. It is possible that the effect of feedback may be moderated by the athletes’ experience and/or performance level. On the other hand, social comparisons allow people to obtain information regarding their ability in relation to others. For Cheng and Lam [61], such social comparisons are a means of self-evaluation and of the development of self-concept. Recent studies involving the use of social comparative feedback with inexperienced participants observed higher levels of perceived competence in those who received positive feedback in comparison with those who received negative or no feedback [32]. Contradicting these findings, the present study has not detected more positive results in perceived competence and autonomous motivation for those in the positive feedback group, although these participants were highly experienced.

With respect to the lower levels of positive affect found in the negative feedback group, two important considerations must be made. First, it is important to mention and that feedback yielded no effect in participants’ negative affect or vitality, the other well-being indicators. Negative feedback generated fewer positive effects, but no increase in negative emotions nor any drop in a general state of aliveness and energy, as vitality is defined [20]. A reduction in positive emotions seems to implicate a smaller effect than an increase in negative emotions or a loss of energy and aliveness. Women expert handball players who received negative feedback were not significantly affected in their well-being apart from fewer physical manifestations in positive emotions after the task. Second, in a previous study with inexperienced players, those who received negative feedback showed lower levels of subjective vitality after a throwing-ball task than those who received positive feedback [32]. In our study, expert players did not see their subjective vitality affected by the negative feedback. Bringing both findings together, we might speculate that expert players could be more protected from the effects of receiving negative feedback than inexperienced players.

In sum, based on the results found, the three types of feedback did not significantly influence the psychological variables in expert players except in the case of positive affect.

With respect to performance variables (throwing speed and accuracy), those who received negative feedback showed a higher throwing speed. No differences in throwing accuracy were found due to feedback. These findings not only differ from studies in which inexperienced participants who received negative feedback performed no worse than those who received positive feedback [28,42], but also from other studies such as Saemi [24] or Ávila [2], in which the positive feedback group in the retention test demonstrated greater accuracy in a throwing task. These results could be related to the athletes’ previous expectations of high performance. In Wulf and Lewthwaite [62,63], it is suggested that information indicating below-average performance triggers thoughts about the self, a mechanism they termed the “self-invoking trigger”. Likewise, previous research focused on the effects of negative feedback [33] serves two important functions (motivates athletes and guides them towards performance improvement). In the case of expert woman handball players, it increases sports performance in the short term.

### Limitations and Future Research

Like all studies, this one also has its limitations. First, the sample size was small. This aspect can cause problems in statistical power which limits the appearance of significant effects, as may have occurred with the performance variables. Future research using a similar design but with a larger number of participants would be beneficial and necessary. On the other hand, the effects of feedback have only been studied here in short-term research. Future studies should investigate the effect of applying different types of feedback over longer periods of time, such as throughout a full season. Likewise, future studies on the effect of feedback should also be developed during a real competition with opponents. This would increase the ecological validity of these type of studies. Participants’ gender could also have played a role in these results, making it necessary to study the effects of feedback on expert men handball players’ behavior. At last, future studies should also address the effect of the feedback provided by the coach in regard to the athletes’ perception of the coach’s competence through psychological variables and performance.

## 5. Conclusions

In light of the results of this study conducted with highly expert women handball players, the three different types of feedback did not generate significant changes in the psychological variables after the task (with the only exception of positive affect). Regarding the performance variables, an increase in the throwing speed was observed in those who received negative feedback.

## Figures and Tables

**Table 1:** Descriptive information of psychological and performance variables.

Performance Variables	Feedback	Set of Pitches	Median	Q1–Q3	Rank Mean	*N*
Throw	f_0_	1–10 p.	88.00	83.08–87.96	48.00	13
Speed		11–20 p.	90.63	88.99–93.23	62.42	13
		21–30 p.	90.63	86.96–92.27	56.42	13
	f_−_	1–10 p.	86.09	82.60–87.69	31.92	13
		11–20 p.	91.73	87.09–94.46	67.35	13
		21–30 p.	92.31	88.12–96.22	74.00	13
	f_+_	1–10 p.	91.20	85.21–93.33	57.58	13
		11–20 p.	92.21	87.94–92.60	62.58	13
		21–30 p.	92.47	90.59–94.52	70.73	13
Throw	f_0_	1–10 p.	45.38	39.23–51.56	54.38	13
Accuracy		11–20 p.	48.68	44.54–58.17	62.46	13
		21–30 p.	41.44	36.13–44.93	44.46	13
	f_−_	1–10 p.	50.93	40.95–55.74	59.62	13
		11–20 p.	39.61	31.99–48.92	45.62	13
		21–30 p.	36.92	33.54–41.87	40.38	13
	f_+_	1–10 p.	51.58	43.76–62.53	69.15	13
		11–20 p.	53.57	37.52–82.12	71.69	13
		21–30 p.	62.15	51.34–70.85	83.23	13
**Psychological Variables**	**Feedback**	**Time of Measure**	**Median**	**Q1–Q3**	**Rank Mean**	***N***
Competence	f_0_	Before	5.75	5.75–6.00	48.35	13
Valuation		After	5.25	5.00–5.50	25.00	13
	f_−_	Before	6.00	6.00–6.00	57.23	13
		After	5.25	5.00–5.50	25.12	13
	f_+_	Before	6.00	5.50–6.00	50.96	13
		After	5.50	5.25–5.50	30.35	13
Perceived	f_0_	Before	4.00	3.40–4.20	38.00	13
Competence		After	3.80	3.20–4.60	35.38	13
	f_−_	Before	4.60	4.00–5.00	49.88	13
		After	4.40	3.20–4.60	35.69	13
	f_+_	Before	4.00	3.00–4.80	37.04	13
		After	4.20	3.40–4.60	41.00	13
Autonomous	f_0_	Before	6.50	5.92–6.67	31.58	13
Motivation		After	6.25	5.83–6.67	31.12	13
	f_−_	Before	6.58	6.17–6.92	42.85	13
		After	6.58	5.75–7.00	41.23	13
	f_+_	Before	6.83	5.83–7.00	43.15	13
		After	7.00	5.75–7.00	47.08	13
Subjective	f_0_	Before	5.67	5.00–5.83	38.85	13
Vitality		After	5.17	4.50–5.83	35.23	13
	f_−_	Before	5.67	4.83–6.00	41.15	13
		After	5.00	3.83–5.67	30.42	13
	f_+_	Before	5.67	5.00–6.50	46.62	13
		After	5.67	4.67–6.83	44.73	13
Positive	f_0_	Before	4.10	3.90–4.30	41.19	13
Affect		After	4.10	3.40–4.30	39.04	13
	f_−_	Before	4.20	3.90–4.30	41.23	13
		After	3.70	3.10–4.10	28.35	13
	f_+_	Before	4.00	3.40–4.70	41.35	13
		After	4.30	3.40–4.80	45.85	13
Negative	f_0_	Before	2.50	1.90–2.70	46.04	13
Affect		After	2.80	1.80–3.30	46.62	13
	f_−_	Before	1.80	1.50–2.30	35.42	13
		After	2.40	1.90–2.90	43.15	13
	f_+_	Before	1.90	1.40–2.80	34.69	13
		After	1.70	1.30–2.70	31.08	13

Note: f_0_ = no-feedback; f_−_ = negative feedback; f_+_ = positive feedback; Q1 = quartile 1; Q3 = quartile 3.

**Table 2:** Non-parametric repeated measures ANOVA-type models of performance variables for feedback, time of measure and their interaction, and post-hoc comparisons.

Performance Variables	Feedback	Time	Feedback*Time	Post-Hoc Comparisons for Significant Interactions (Cliff’s Delta ^b^)					
	F (df ^a^)	F (df ^a^)	F (df ^a^)	No Feedback f_0_	Negative Feedback f_−_	Positive Feedback f_+_	1–10 Pitches t_0_	11–20 Pitches t_1_	21–30 Pitches t_2_
Throw	0.29	11.48 ***	3.06 *	t_0_ = t_1_ (−0.25, S)	t_0_ < t_1_ (−0.57, L) ***	t_0_ = t_1_ (−0.02, N)	f_0_ = f_−_ (−0.23, S)	f_0_ = f_−_ (0.15, S)	f_0_ = f_−_ (0.28, S)
Speed	(1.98)	(1.83)	(3.42)	t_1_ = t_2_ (0.14, N)	t_1_ = t_2_ (−0.15, S)	t_1_ = t_2_ (−0.21, S)	f_0_ = f_+_ (−0.2, N)	f_0_ = f_+_ (0.08, N)	f_0_ < f_+_ (−0.30, S)
				t_0_ = t_2_ (0.21, S)	t_0_ < t_2_ (−0.64, L) ***	t_0_ = t_2_ (−0.16, S)	f_−_ = f_+_ (−0.30, S)	f_−_ = f_+_ (0.10, N)	f_−_ < f_+_ (0.12, N)
Throw	4.19 *	0.41	2.10						
Accuracy	(1.82)	(1.87)	(3.44)						

^a^ The denominator of all df values is ∞; e.g., 1.96, ∞. ^b^ Cliff’s Delta interpretation: N = negligible, S = small, M = medium, L = large; α-level is set at 0.05; * *p* < 0.05, *** *p* < 0.001.

**Table 3:** Non-parametric repeated measures ANOVA-type models of psychological variables feedback, time of measure and their interaction, and post-hoc comparisons.

Psychological Variables	Feedback	Time	Feedback*Time	Post-Hoc Comparisons for Significant Interactions (Cliff’s Delta ^b^)				
	F (df ^a^)	F (df ^a^)	F (df ^a^)	No Feedback f_0_	Negative Feedback f_−_	Positive Feedback f_+_	Before Throwing Task t_0_	After Throwing Task t_1_
Competence	0.32	68.31 ***	1.28					
Valuation	(1.97)	(1.00)	(1.96)					
Perceived	0.30	1.59	2.44					
Competence	(1.98)	(1.00)	(1.84)					
Autonomous	1.48	0.09	0.64					
Motivation	(1.74)	(1.00)	(1.70)					
Subjective	0.90	2.99	0.75					
Vitality	(1.80)	(1.00)	(1.77)					
Positive	0.55	2.23	4.63 *	t_0_ = t_1_ (0.07, N)	t_0_ > t_1_ (0.36, M) **	t_0_ = t_1_ (−0.09, N)	f_0_ = f_−_ (0.03, N)	f_0_ = f_−_ (−0.30, S)
Affect	(1.85)	(1.00)	(1.75)				f_0_ = f_+_ (0.01, N)	f_0_ = f_+_ (−0.18, S)
Negative	1.32	0.38	1.70					
Affect	(1.95)	(1.00)	(1.94)					

^a^ The denominator of all df values is ∞; e.g., 1.96, ∞. ^b^ Cliff’s Delta interpretation: N = negligible, S = small, M = medium, L = large; α-level is set at 0.05; * *p* < 0.05, ** *p* < 0.01, *** *p* < 0.001.
